# Forensic Medicine

**Published:** 1837-01

**Authors:** 


### FORENSIC MEDICINE.

The following trial, which occurred at the Perth Assizes, is of some medico-legal interest.

It was proved in evidence that a middle-aged man, of intemperate habits, whilst intoxicated and lying on the floor, was struck twice on the left shin with a poker; that after this he was lifted up, and seated on a chair; that he then limped to the door, a distance of fifteen feet; that his leg was caught between the leaves of the door as he went out; that almost immediately afterwards he was found sitting on the smooth flag stones before the door, with the left tibia projecting three or four inches through a wound in the integuments; that he became delirious soon afterwards, and died at the end of a fortnight. On dissection, the tibia was found fractured above the middle, with small detached splinters, and the bone dead to the extent of an inch on both sides of the injury, being quite smooth and ivory looking, and surrounded by an effusion of new bone, between which and the new portion an ulcerated groove was forming. A comminuted fracture of the head of the fibula was discovered on dissection, which had been overlooked.

Professor Syme was examined. He considered the fracture of the tibia had been caused by a violent blow, and could not have been produced by a simple fall of the body, without impulse. He thought so from the part fractured, the direction, and the dead bone. He thought the deceased might have walked to the door after the tibia was fractured, and, though very improbable, it was not impossible after the fibula also was broken. The slamming of the door would account for the fracture of the fibula: no ecchymosis would have appeared for several hours after the injury. Where the tibia protruded there would be no ecchymosis, as it would give exit to the blood. An ecchymosis over the tibia might escape notice.

For the defence it was contended, that the fracture had been occasioned by the deceased falling on the smooth stones at his own door; that the tibia had been fractured alone by the fall; and that the head of the fibula had been broken by "rough handling" after death; that, if the fracture had been caused by external violence, there would have been ecchymosis; that the deceased could not have walked a few steps after the fracture, even supposing the fibula had remained entire, and that the dead appearance of the bone could be accounted for by its protrusion, or subsequent gangrene.

The jury decided that the fracture had not been caused by external violence.

Professor Syme makes the following remarks on the trial: 1. He does not believe that a simple fall on a level surface could cause a compound fracture of the leg; nor that the tibia, if broken in this way alone, the fibula remaining entire, could project three or four inches from the wound. 2. Besides other obvious objections, the explanation that the fibula was broken by rough handling, requires it to be believed that the fibula remained sound whilst the broken end of the tibia projected three or four inches, which is impossible. The following cases of recent occurrence best answer the questions whether it is impossible to walk a few steps after fracture of the tibia, and whether the presence of ecchymosis is a necessary or never-absent consequence of compound fracture caused by external violence:—Dr. L., of Strasbourg, while travelling in the Highlands, broke both the bones of the leg, and afterwards travelled 170 miles by a variety of conveyances to put himself under Mr. Syme's care. On his arrival, the third day after the accident, he went into a warm bath without any bandage on the limb.—Mr. Crichton, of Dundee, had a patient who, under the influence of delirium tremens, danced about the room, with both bones fractured near the ankle, and protruding through the integuments. Mrs. M., æt. forty-eight, was admitted with compound and comminuted fracture of the left leg, from a wheel passing over it: the wheel had also passed over the thigh, producing considerable ecchymosis, but there was no ecchymosis at the seat of the fracture. Many pieces of bone exfoliated. This case illustrates Mr. Syme's remarks on ecchymosis, and on the exfoliation of bone after external violence.

*Edinburgh Medical and Surgical Journal, October,* 1836.

